# Exploratory Investigation of Barriers to Writing Case Reports Among Japanese Physicians Who Participated in Web Study Sessions

**DOI:** 10.7759/cureus.81857

**Published:** 2025-04-07

**Authors:** Tsuneaki Kenzaka, Shinsuke Yahata, Naoya Mizutani, Ayako Kumabe, Ryo Fujiwara, Koki Kosami, Hiroyuki Mori

**Affiliations:** 1 Department of Internal Medicine, Hyogo Prefectural Tamba Medical Center, Tamba, JPN; 2 Division of Community Medicine and Career Development, Kobe University Graduate School of Medicine, Kobe, JPN; 3 Department of General Internal Medicine, Hyogo Prefectural Harima-Himeji General Medical Center, Himeji, JPN; 4 Department of General Medicine, Toyooka Public Hospital, Toyooka, JPN; 5 Division of Public Health, Center for Community Medicine, Jichi Medical University, Shimotsuke, JPN

**Keywords:** barriers, clinical message, trainees, trainers, writing case reports

## Abstract

Objective: This study examined the barriers to writing case reports among Japanese physicians who participated in a web study series titled “Let’s Write a Case Report! What you need to do to be Accepted” and explored differences in these barriers based on physicians’ years of experience.

Methods: The study design was cross-sectional in nature. Throughout August and November 2020, the respondents participated in the web study sessions. Using an 11-point scale, from -5 (not a barrier at all) to 0 (neither) to 5 (very much of a barrier), the 16 items interrogated which elements could be barriers to case report writing. Participants were divided into two groups: those with 10 or fewer years of postgraduation experience and those with 11 or more years of postgraduation experience. The basic attributes and possible barriers were compared between the groups using chi-squared and t-tests.

Results: In total, 53 (62.4%) participants responded to the web-based questionnaire. In a comparison between the two groups, the following barriers were significantly higher in the 10 years or fewer group: having sufficient medical documentation to write a report, knowing how to write a case report, determining the main case points and clinical message, knowing how to search the literature, knowing how to obtain literature, meeting the financial cost of accessing literature. Although there were no significant differences, only the lack of motivation to write papers was perceived as a barrier by the 11 years or more group.

Conclusions: Determining the main case points and clinical message, selecting an appropriate journal for submission, and having adequate time to write a paper were the major barriers to writing a case report. Paper-writing motivation was crucial for those with longer clinical experience and experience in writing papers. These barriers were significantly more pronounced among those with fewer than 10 years of clinical experience than among those with 11 or more years of experience. Providing targeted support in developing case points and clinical messages will lead to an increase in the rate of case report writing.

## Introduction

Writing case reports is considered highly important for all physicians, including general practitioners, in terms of contributing to the academic field [1] and improving their clinical skills [2]. However, it has been reported that only 3.8% of the abstracts presented at the Japan Primary Care Association Annual Meetings are published afterward [3]. Furthermore, only a small percentage of these abstracts are published as case reports [4]. Several barriers to writing case reports among Japanese generalists have been reported, including challenges in the writing process, literature search/accessing articles, time required to write articles, reluctance to write articles in English, and limited options for suitable journals [4].

We have been conducting regular study sessions on case report writing to improve the rate of paper preparation. Most participants are physicians who are interested in writing articles but seek guidance on how to do so effectively, as well as instructors who wish to teach young physicians how to write. Participants belong to various medical departments and are not limited to those in general practice.

Barriers to case report writing have not been examined extensively [4]. In particular, the different barriers to case report writing based on physicians’ years of experience remain understudied. Therefore, the present study aimed to (1) examine barriers to writing case reports among the participants and (2) identify differences in barriers based on physicians’ years of experience.

## Materials and methods

This study was an exploratory descriptive study and was approved by the Ethics Committee of Hyogo Prefectural Tamba Medical Center (approval number: Tan-I number 1304). The respondents were physicians who participated in web study sessions titled “Let’s Write a Case Report! What you need to do to be Accepted,” held in August and November 2020. Physicians who agreed to participate and provided informed consent for the questionnaire were included in the study. The exclusion criteria were medical students and other nonphysicians. As this was an exploratory study, no specific sample size was determined in advance.

The case report writing sessions are conducted as follows. Several times a year, we hold study sessions to support physicians in writing case reports. Participants are recruited through the mailing lists of the Japan Primary Care Association, Japanese Hospitalists, and a case report writing group on a social networking service. The sessions are held online, and each session lasts 120-150 minutes.

Each session includes a 30-minute lecture covering the key points and common pitfalls in writing a case report. This is followed by participants discussing how to write a case report using an actual case, with one of the organizers facilitating the discussion. For cases that have already been published, the publication process is explained. Participants who aim to publish a case report in the future may also present their own cases; following the discussion, the organizers provide suggestions regarding how the papers can be published. If requested by participants, organizers continue to support participants until their papers are published.

We administered a web-based questionnaire to the participants, with questions about sex, age, number of years since graduation, medical department, prior experience with writing case reports, and, if applicable, the number of case reports they had written. Furthermore, the questionnaire included items about 16 factors identified in a previous study [4] as potential barriers to writing case reports.

Participants were asked to rate each item using an 11-point scale from -5 (not a barrier at all) to 0 (neither) to 5 (very much of a barrier). This scale was designed with a midpoint of 0 to improve visualization and make it easier for respondents to answer, in contrast to a typical 0-10 Likert scale.

The items addressed the specific following barriers to writing case reports: (1) recognizing cases suitable for a case report; (2) having sufficient medical documentation to write a report; (3) knowing how to write a case report; (4) determining the main case points and clinical message; (5) lacking a mentor or supporter; (6) knowing how to search the literature; (7) knowing how to obtain literature; (8) meeting the financial cost of accessing literature; (9) meeting the cost of proofreading; (10) meeting the cost of publication; (11) having adequate time to write; (12) lacking motivation to write; (13) experiencing difficulty with English; (14) selecting an appropriate journal for submission; (15) determining whether ethical review is required; and (16) knowing how to apply for ethical review.

To analyze the data, responses to these questions were tabulated. In previous Japan-based studies, students and physicians have usually been regarded as trainees for up to 10 years following postgraduation [5,6]. Therefore, we classified the participants into two groups: those who had graduated within the past 10 years (trainees) and those who had been trainees for 11 years or more (trainers).

Additionally, the basic attributes and possible barriers were compared between the groups using chi-squared and t-tests (p < 0.05 indicates a significant difference). All statistical analyses were performed using Stata MP, version 15 (StataCorp College Station, College Station, TX, USA).

## Results

Of the 85 participants, 51 (60.0%) completed the web-based questionnaire. In total, 37 (72.5%) patients were males and 14 were females (27.5%). The mean age ± standard deviation was 36.9 ± 10.0 years. Participants’ years of postgraduation experience ranged from one to 33 years (median: 10 years). Among those who completed the questionnaire, 27 (52.9%) had experience writing case reports. Among this subgroup, the number of case reports written ranged from one to eight (median: 2). Table 1 shows the distribution of basic attributes between the two groups. The group with more than 11 years of postgraduation experience had significantly more experience writing case reports.

**Table 1:** Distribution of basic attributes between the two groups

	10 or fewer years of postgraduation experience group (n = 28)	11 or more years of postgraduation experience group (n = 23)	p-value
Male, n (%)	197 (60.7%）	20 (87.0%)	0.058
Age (years, mean ± standard deviation)	29.7 ± 3.4	45.8 ± 7.8	<0.001
Experience in writing case reports, n (%)	8 (28.6%）	19 (82.6%)	<0.001

Table 2 presents the physicians’ departmental affiliations. Participants belonged to various medical departments and were not limited to general practice.

**Table 2:** Physician department affiliations among the groups

	All participants (N = 51)	10 or less years of postgraduation experience group (N = 28)	11 or more years of postgraduation experience group (N = 23)
	n (%)	n (%)	n (%)
General medicine	9 (17.6%)	6 (21.4%)	3 (13.0%)
Internal medicine	13 (25.5%)	6 (21.4%)	7 (30.4%)
Neurology	0 (0.0%)	0 (0.0%)	0 (0.0%)
Respiratory medicine	2 (3.9%)	1 (3.5%)	1 (4.3%)
Gastroenterology	1 (2.0%)	0 (0.0%)	1 (4.3%)
Cardiology	0 (0.0%)	0 (0.0%)	0 (0.0%)
Allergology	0 (0.0%)	0 (0.0%)	0 (0.0%)
Rheumatoid collagen disease	1 (2.0%)	1 (3.5%)	0 (0.0%)
Diabetes medicine	0 (0.0%)	0 (0.0%)	0 (0.0%)
Endocrinology	0 (0.0%)	0 (0.0%)	0 (0.0%)
Nephrology	0 (0.0%)	0 (0.0%)	0 (0.0%)
Hematology	1 (2.0%)	0 (0.0%)	1 (4.3%)
Gastroenterology	0 (0.0%)	0 (0.0%)	0 (0.0%)
Emergency department	4 (7.8%)	3 (10.7%)	1 (4.3%)
Pediatrics	5 (9.8%)	0 (0.0%)	5 (21.7%)
Gynecology	1 (2.0%)	1 (3.5%)	0 (0.0%)
Surgical department	1 (2.0%)	0 (0.0%)	1 (4.3%)
Infectious diseases	2 (3.9%)	2 (7.1%)	0 (0.0%)
Anesthesiology	1 (2.0%)	0 (0.0%)	1 (4.3%)
Initial resident	5 (9.8%)	5 (17.8%)	0 (0.0%)
No answer	5 (9.8%)	3 (10.7%)	2 (8.7%)

Table 3 illustrates the different factors considered to be barriers to case report writing and the degree to which they were perceived as such. Overall, the main barriers were recognizing cases suitable for a case report, knowing how to write a case report, determining the main case points and clinical message, lacking a mentor or supporter, having adequate time to write, and selecting an appropriate journal for submission.

**Table 3:** Results of the factors and the degree of barriers to writing case reports

	All participants	10 or less years of postgraduation experience group	11 or more years of postgraduation experience group	
	Mean ± standard deviation	Mean ± standard deviation	Mean ± standard deviation	p-values
Barrier factors questions				
Recognizing cases suitable for a case report	1.66 ± 1.90	2.07 ± 1.78	1.26± 2.03	0.135
Having sufficient medical documentation to write a report	1.41 ± 2.02	1.96 ± 1.87	0.78 ± 2.11	0.039
Knowing how to write a case report	1.64 ± 2.22	2.29 ± 2.11	0.96 ± 2.25	0.034
Determining the main case points and clinical message	2.43 ± 2.09	3.07 ± 1.76	1.78 ± 2.30	0.028
Lacking a mentor or supporter	1.60 ± 2.35	1.75 ± 2.49	1.57 ± 2.27	0.783
Knowing how to search the literature	0.25 ± 2.47	0.86 ± 2.17	-0.65 ± 2.64	0.030
Knowing how to obtain literature	0.00 ± 2.67	1.00 ± 2.35	-1.30 ± 2.53	0.002
Meeting the financial cost of accessing literature	0.06 ± 2.80	1.07 ± 2.57	-1.30 ± 2.62	0.002
Meeting the cost of proofreading	0.55 ± 2.63	1.14± 2.51	-0.13 ± 2.76	0.091
Meeting the cost of publication	0.74 ± 2.73	1.00 ± 2.78	0.35 ± 2.76	0.406
Having adequate time to write	2.09 ± 2.20	2.46 ± 2.17	1.74 ± 2.28	0.251
Lacking motivation to write	0.79 ± 2.52	0.54 ± 2.80	1.22 ± 2.24	0.254
Experiencing difficulty with English	1.17 ± 2.58	1.50 ± 2.47	1.04 ± 2.53	0.519
Selecting an appropriate journal for submission	2.15 ± 1.84	2.46 ± 1.92	1.91 ± 1.76	0.294
Determining whether ethical review is required	0.94 ± 2.55	1.14 ± 2.61	0.74 ± 2.62	0.585
Knowing how to apply for ethical review	0.87 ± 2.46	1.25 ± 2.34	0.43 ± 2.61	0.251

When comparing the two groups, the following barriers were significantly more prominent in the 10 years or fewer postgraduation experience group: having sufficient medical documentation to write a report, knowing how to write a case report, determining the main case points and clinical message, knowing how to search the literature, knowing how to obtain literature, and meeting the financial cost of accessing literature. Although not statistically significant, lacking motivation to write papers was the most prominent factor perceived as a barrier by the 11 years or more group compared to the 10 years or fewer group.

## Discussion

The participants in this study were mainly individuals who wished to write a case report but were unsure how to proceed, as well as those interested in teaching others how to write case reports. Very few had enough experience to write a case report independently.

Barriers to case report writing identified among Japanese generalists [4] were as follows: recognizing cases suitable for a case report, having sufficient medical documentation to write a report, knowing how to write a case report, determining the main case points and clinical message, lacking a mentor or supporter, knowing how to search the literature, knowing how to obtain literature, having adequate time to write, and lacking motivation to write. The factors previously identified as barriers to case report writing for Japanese generalists [4] were mostly also reported in this study as barriers for physicians, including nongeneralists, albeit to varying degrees. Determining the main case points and clinical message was the most important barrier. Having adequate time to write a paper and selecting an appropriate journal for submission also scored high as barriers. Other main barriers were the paper writing method, recognizing cases suitable for a case report, knowing how to write a case report, and lacking a mentor or supporter. These results are similar to those of a survey conducted at a workshop for members of the Japan Primary Care Association [4]. As mentioned in the tips for case report writing [7], the most important issue is creating a novel perspective or clinical message.

The 10 years or fewer group was significantly more likely than the 11 years or more group to have experienced the following barriers: having sufficient medical documentation to write a report, knowing how to write a case report, determining the main case points and clinical message, knowing how to search the literature, knowing how to obtain literature, and meeting the financial cost of accessing literature. In contrast, the group with 11 years or more of postgraduation experience had significantly more experience in both academic writing and clinical experience. While both groups had similar perceived barriers, the extent to which these barriers were perceived differed significantly between them.

Conversely, although the difference was not statistically significant, only lacking motivation to write papers was perceived as the most prominent by the group with 11 or more years of postgraduation experience, compared to the 10 years or fewer group. These results suggest that it is necessary to motivate those who have longer clinical and paper-writing experience to proactively write papers.

In a study targeting young nephrologists in India [8], major barriers to writing papers, not limited to case reports, were reported to be time available to write a paper (82.1%), funding (67.9%), and limited access to research articles (65.4%). Time constraints were similar to those in the present study, but in this study, money was not such a strong barrier. Barriers may differ depending on the country in which the doctor works, years of experience, or the department in which the doctor practices; future research should explore this topic. The average monthly income of a doctor in Japan is estimated to be 700,000 JPY (INR 3.94 lakhs) [9], whereas in India, it is estimated to be up to about INR 1.0 lakhs [10]. Given that publication fees for open-access international journals are generally consistent across countries, we believe that income disparity may account for the difference in the impact of financial barriers on writing papers.

This study has several limitations. The target population comprised participants in the case report study group, whose evident interest in case report writing limits their representativeness for generalization purposes. Moreover, the importance of determining the main case points and clinical messages was strongly emphasized during the study group lectures, which may have influenced the survey results. In this study, 71.4% of trainees and 17.4% of trainers had never written a case report. Financial concerns can be a barrier during the actual writing stage; however, they may not be perceived as a barrier at the prewriting stage and could therefore be underestimated. The questionnaire response rate in this study was 60%. A previous study reported a similar survey response rate, also in the 60% range, for lecture participants in Japanese [6]. These studies had comparable sample sizes as well. While we believe this rate is reasonable, the findings may not necessarily reflect the views of all potential participants.

Future research should aim to include a broader range of participants who are less associated with this study group, for example, members of academic societies within specific medical specialties.

## Conclusions

Determining the main case points and clinical message, selecting an appropriate journal for submission, and having adequate time to write a paper were identified as the major barriers to writing a case report. These barriers were significantly more pronounced among those with fewer than 10 years of clinical experience than among those with 11 or more years of experience. Therefore, for those with more clinical and academic writing experience, maintaining motivation to write is considered very necessary. We believe that providing targeted support in identifying the main case points and clinical message for publication may help increase the frequency of case report submissions.
